# Tools for successful proliferation: diverse strategies of nutrient acquisition by a benthic cyanobacterium

**DOI:** 10.1038/s41396-020-0676-5

**Published:** 2020-05-18

**Authors:** H. S. Tee, D. Waite, L. Payne, M. Middleditch, S. Wood, K. M. Handley

**Affiliations:** 1 School of Biological Sciences, University of Auckland, Auckland, New Zealand; 2 Cawthron Institute, Nelson, New Zealand

**Keywords:** Biofilms, Metagenomics

## Abstract

Freshwater cyanobacterial blooms have increased worldwide, channeling organic carbon into these systems, and threatening animal health through the production of cyanotoxins. Both toxic and nontoxic *Microcoleus* proliferations usually occur when there are moderate concentrations of dissolved inorganic nitrogen, but when phosphorus is scarce. In order to understand how *Microcoleus* establishes thick biofilms (or mats) on riverbeds under phosphorus-limiting conditions, we collected *Microcoleus*-dominated biofilms over a 19-day proliferation event for proteogenomics. A single pair of nitrogen-dependent *Microcoleus* species were consistently present in relatively high abundance, although each followed a unique metabolic trajectory. Neither possessed anatoxin gene clusters, and only very low concentrations of anatoxins (~2 µg kg^−1^) were detected, likely originating from rarer *Microcoleus* species also present. Proteome allocations were dominated by photosynthesizing cyanobacteria and diatoms, and data indicate biomass was actively recycled by *Bacteroidetes* and *Myxococcales*. *Microcoleus* likely acquired nutrients throughout the proliferation event by uptake of nitrate, urea, and inorganic and organic phosphorus. Both species also harbored genes that could be used for inorganic phosphate solubilization with pyrroloquinoline quinone cofactors produced by cohabiting *Proteobacteria*. Results indicate that *Microcoleus* are equipped with diverse mechanisms for nitrogen and phosphorus acquisition, enabling them to proliferate and out-compete others in low-phosphorus waters.

## Introduction

Over the past few decades, the occurrence of freshwater cyanobacterial blooms has increased worldwide, and is associated with elevated water temperatures and nutrient enrichment [1–3]. The overgrowth and decomposition of cyanobacterial cells drive excessive oxygen consumption in the aquatic environment [4], and some cyanobacteria produce cyanotoxins, which include potent neurotoxins, cytotoxins, hepatotoxins, and endotoxins [5]. All of these toxins represent a health risk to humans and animals. While most research has focused on planktonic blooms, benthic mat proliferations of toxin-producing cyanobacteria have been reported with increasing frequency [2, 6–8]. In freshwater systems, these benthic proliferations form visible mats that coat the beds of ponds, lakes, and rivers [2, 6, 9, 10]. These mats can be readily ingested by animals, and globally many cases of canine death have been linked to the consumption of benthic mats containing anatoxin-producing cyanobacteria [5, 11, 12], such as *Microcoleus autumnalis* and *favosus* (formerly *Phormidium autumnale* and *favosus*) [13–16].

Cohesive, flow-tolerant, *Microcoleus* mats are found in freshwater systems throughout the world, where they commonly form millimeter-thick mats on riverbeds [2, 13, 17, 18]. *Microcoleus* species that are known to be toxic produce anatoxin-a, its homolog homoanatoxin-a, and their dihydro variants: dihydroanatoxin-a and dihydrohomoanatoxin-a [13, 17]. Acute exposure to these neurotoxins can cause muscular twitching, loss of coordination, and rapid death from respiratory paralysis [19]. Benthic proliferations of *Microcoleus* often comprise mixtures of anatoxin-producing and non-anatoxin-producing species/strains [20]. The relative abundances of these species/strains are thought to be the main determinant of the level of toxins in a biofilm [20].

*Microcoleus* species studied to-date lack nitrogenase genes [18, 21], and their proliferations are associated with dissolved inorganic nitrogen (DIN) enrichment [2, 22]. Contrary to the paradigm that influxes of phosphorus drive eutrophication in freshwater [23], *Microcoleus* readily proliferates in water with low levels of dissolved reactive phosphorus (DRP) [2]. Despite low external phosphorus concentrations, well-established mats are highly enriched in DRP [24]. This internal pool of bioavailable phosphorus is thought to be partly derived from large quantities of fine sediment trapped within the mats. Phosphorus release from this material likely occurs due to high daytime pHs (>9) generated by photosynthesis, and also due to low nighttime dissolved oxygen concentrations (<4 mg L^−1^), resulting from respiration [24]. In addition, alkaline phosphatase activity in *M. autumnalis* cultures suggests they have the capability to mineralize organic phosphorus [25]. However, it is unclear whether indirect mechanisms for phosphorus release, such as high pH, are effective at early proliferation stages when biofilms are extremely thin and patchy, or the extent to which *Microcoleus* mats acquire phosphorus through other routes, such via enzymatic solubilization of inorganic phosphorus or phosphatase-based scavenging of organic phosphorus.

We predicted that to successfully proliferate in low DRP rivers, *Microcoleus* species are capable of scavenging diverse sources of phosphorus, and some of this phosphorus is sourced through interactions with the wider biofilm community. We sampled *Microcoleus* mats from a river to capture temporal trends throughout a benthic proliferation event. We then reconstructed bacterial and protist genomes from the biofilm community, and combined these with metaproteomics to determine taxon-specific metabolic profiles.

## Materials and methods

### Mat growth and sampling

At the onset of a late summer proliferation (4th March 2016), cobbles from the Wai-iti River (Nelson, New Zealand) were removed, cleared of incipient growth with sterile sponges, and placed back into the river. Clearing was gentle as seeding from the pre-existing rock surface is important for mat establishment [26]. Five pre-cleared cobbles were collected at each of three time points to capture the first 3, 6, and 9 days of growth (Table S1). Additional cobbles that contemporaneously developed biofilms were collected at days 12 and 19. Final sampling on 23^rd^ March 2016 was undertaken prior to forecast heavy rain (Fig. S1), which tends to detach thick *Microcoleus* mats. The visible biofilms were collected from the rocks with sterile forceps and preserved upon collection in LifeGuard (Qiagen, Hilden, Germany), and stored at −80 °C.

### Physicochemical, nutrient and toxin analysis

Water temperature and light intensity data were measured every 30 min (HOBO Pendant^®^ UA-002-64, Onset, MA, USA). Daily rainfall and river discharge data were provided by the Tasman District Council, New Zealand. Analysis of nitrite-N, nitrate-N, ammoniacal-N and DRP were carried out using a flow injection analyzer, following a modified APHA 4500 protocol [27]. Anatoxin-a, homoanatoxin-a, dihydroanatoxin-a, and dihydrohomoanatoxin-a were measured using LC-MS/MS [15]. Briefly, biofilm samples were lyophilized and 1 mL of Milli-Q water with 0.1% formic acid. Compounds were then separated by liquid chromatography (Waters Acquity UPLC, Waters Corp., MA, USA) on a BEH C18 column (1.7 μm, 1 × 50 mm, Waters Corp., MA, USA) and quantified on a Quattro Premier XE triple quadrupole mass spectrometer (Waters-Micromass, Manchester, UK).

### Brightfield microscopy

Biofilms were washed once with 1× Phosphate Buffer Saline and imaged with a Leica DMR upright microscope using LAS X imaging software (Leica, Germany).

### DNA extraction and sequencing

DNA was extracted from 0.02–0.14 g of each sample using a MoBio Power Soil DNA Kit (MoBio, CA, USA) and prepared for 16S rRNA amplicon and whole genome shotgun (WGS) sequencing. PCR amplification of 16S rRNA genes was performed using modified Earth Microbiome Project primers EMP-16S-515F [28] and EMP-16S-806R [29]. Reactions (50 µL) were carried out using JumpStart REDTaq Readymix (Sigma-Aldrich, MO, USA) with an initial denaturation step of 95 °C for 5 min, 35 cycles of 95 °C for 45 s, 50 °C for 60 s, 72 °C for 90 s, and a final extension step of 72 °C for 10 mins. The resulting amplicons were purified with Agencourt AMPure XP magnetic beads (Beckman Coulter, CA, USA). Library preparation and 2 × 250 bp sequencing was performed using the Illumina MiSeq platform with V2 chemistry at Auckland Genomics (University of Auckland, NZ). For WGS sequencing, a minimum of 250 ng of DNA was used. TruSeq DNA nano libraries were prepared to create 550 bp fragments for 2 × 250 bp sequencing using the Illumina HiSeq 2500 platform with V2 chemistry at the Otago Genomics Facility (University of Otago, NZ).

### Amplicon analysis

Raw sequences were merged using USEARCH 9.0 -fastq_mergepairs [30] and filtered using sickle [31] with a minimum Phred score of 30. Sequences were clustered based on a similarity threshold of ≥99.9% into 913 operational taxonomic units (OTUs) using the UCLUST pipeline [32]. OTUs were classified using the USEARCH 9.0 global alignment algorithm with the SILVA SSU Ref NR 99 132 [33]. Non-prokaryotic sequences were removed and sequences were rarefied to 2 870 using QIIME2 2019.4 [34] leaving 643 OTUs. The Shannon-Wiener index from the BiodiversityR [35] package in R version 3.5.1 [36] was used to measure bacterial alpha diversity. Nonmetric multidimensional scaling (NMDS) ordinations were plotted based on Bray-Curtis dissimilarities, environmental variables were fitted and *p* values were generated using the vegan package metaMDS and envfit function [37] with R version 3.5.1 [36].

### Small subunit (SSU) rRNA gene reconstruction and genome assembly

WGS reads were trimmed using sickle [31] with a minimum Phred score of 30, generating 285 million paired-end reads. Full length 16S rRNA sequences were reconstructed from the combined dataset over 50 iterations using EMIRGE [38] with the SILVA SSU Ref NR 99 132 database, and a clustering threshold of 100%, resulting in 201 sequences. For genome assembly, trimmed reads from the same timepoints were coassembled using metaSPAdes [39] with *k-mer* values 41, 61, 81, 101, 127.

### Genome binning and annotation

The 152 799 assembled scaffolds (≥2 kb in length) were binned using MetaBAT [40], CONCOCT [41] and MaxBin 2.0 [42]. The highest quality prokaryotic bins from each assembly were selected using DASTool [43]. Bins occurring at more than one timepoint were dereplicated using dRep with default parameters, such that bins sharing ≥99% average nucleotide identity (ANI) were dereplicated [44]. Eukaryotic bins were identified according to methods described in Supplementary Materials. The final set of dereplicated prokaryotic and eukaryotic bins was validated using VizBin [45] by calculating tetranucleotide frequencies with the Barnes-Hut stochastic neighbor embedding algorithm. Eighty-two bins were recovered (Table S2), including a combined bin of diatom genomes and 81 prokaryote bins (CheckM completeness >75%). Of these, 45 were near-complete (CheckM completeness >95%) [46]. Genome coverage was determined by mapping reads to bins using Bowtie version 1.2.0 [47], allowing ≤3 mismatch. Open Reading Frames were predicted and protein sequences predicted using Prodigal [48]. Genes were annotated by searching against UniProt [49], UniRef100 [50], and KEGG Orthologous groups [51] databases using USEARCH 9.0 [30] with an e-value cutoff of 0.001 and a minimum of 50% identity required. Sequences were also searched against TIGRFAMs [52] and Pfam [53] using HMMER3 [54] with an e-value cutoff of 0.001, and only hits with the lowest e-value were retained. Taxonomy was assigned using the Genome Taxonomy Database Toolkit (GTDB-TK) v1.3 with release r86 [55].

### Growth rates

Genome replication rates were determined for metagenome-assembled genomes (MAGs) with coverage ≥5, completeness ≥75%, and ≤175 scaffolds per Mbp of sequence [56]. Read mapping files were used to calculate the index of replication using iRep default parameters and without GC correction [56]. These MAGs were also used to estimate minimum generation times using growthpred [57] with parameters “-b -c 0 -r –T 20 -S”. The predicted minimum generation time in this study was calculated based on the organisms synonymous codon usage bias from a set of normally highly expressed genes (mainly rRNA, tRNA) and their growth temperature [57].

### Cyanobacteria phylogeny

EMIRGE-reconstructed cyanobacterial 16S rRNA gene sequences were aligned to the SILVA 132 database [38] using SINA (http://www.arb-silva.de/aligner). Poorly aligned sequences were filtered using MOTHUR [58]. A tree was computed with RAxML using the maximum likelihood method with a GTRGAMMA model and 100 bootstraps [59]. A set of 88 core marker protein sequences were identified, concatenated and aligned using GTDB-tk [55] from cyanobacterial MAGs and closely related genomes from the NCBI Genome database [60] (Table S3). The phylogenetic tree was inferred using RAxML [59] with the PROTGAMMAAUTO model and 100 bootstraps. Trees were annotated and visualized using iTOL [61].

### Protein extraction and detection

Biofilms were freeze-dried and suspended in 100 µL of lysis buffer (7 M Urea; 2 M Thiourea; 10 mM DTT; 50 mM Ammonium Bicarbonate, pH 8.0) per 1 mg dry weight of sample. The solution was homogenized through sonication on ice. Protein concentrations were determined using the EZQ fluorescent protein assay (Invitrogen, CA, USA). Aliquots containing 100 µg of protein were adjusted to 100 µL with lysis buffer, and proteins were reduced by incubating at 56˚C for 1 h. Proteins were alkylated by adding iodoacetamide (final concentration 50 mM) and incubating in the dark for 30 min at room temperature. Reactions were quenched by addition of DTT (final concentration 20 mM). Reduced and alkylated proteins were diluted in 9 volumes of 50 mM Ammonium Bicarbonate, pH 8.0, and 3 µg of trypsin was added before samples were digested overnight at 37 ˚C. Digested proteins were acidified and desalted using HLB columns (Waters Corp, MA, USA) and eluted peptides dried in a Savant Speedvac concentrator (Thermo Fisher Scientific, MA, USA). Peptides were resuspended in 0.5 M triethylammonium bicarbonate (TEAB). Aliquots were taken from a representative subset of samples to create a reference pool for normalization of iTRAQ signals across different runs before samples and the reference pool were labeled with iTRAQ 8-plex isobaric tags (Sciex, MA, USA). Labeled samples were pooled and desalted on an HLB column, and analyzed on a TripleTOF 6600 Quadrupole-Time-of-Flight mass spectrometer (Sciex) coupled to an Eksigent NanoLC 400 UHPLC system, controlled using Analyst TF 1.7 software (Sciex) (see Supplementary Materials for run parameters).

### Metaproteome analysis

Peptides were identified using ProteinPilot v5.0 software (Sciex, MA, USA) and predicted protein sequences from the dereplicated set of MAGs. Search parameters were: Sample Type, iTRAQ 8-plex (Peptide Labelled); Search Effort, Thorough; Cys Alkylation, Iodoacetamide; Digestion, Trypsin. A total of 671 proteins were confidently identified and quantified based on the following criteria: (i) local False Discovery Rate <5%, (ii) total unused score >1.3 (95% CI), and (iii) proteins were identified in at least two out of three biological replicates. The geometric mean and standard deviation were calculated for replicates. To account for changes in taxa relative abundances, proteins were also normalized to genome coverage. Log fold-change was calculated by subtracting the initial log-transformed value from the final log-transformed value i.e., log fold-change = log_10_(final)–log_10_(initial) = log_10_(final/initial). Heatmaps were plotted using gplots [62] in R version 3.5.1 with row-wise scaling for comparison of log-transformed protein expression across timepoints.

### Database submissions

All sequence data from this study have been deposited with NCBI under bioproject PRJNA555798. Peptide and protein summary files were exported to the PRoteomics IDEntifications database under the identifier PXD015625.

## Results and discussion

### *Microcoleus* growth conditions

**Fig. 1:**
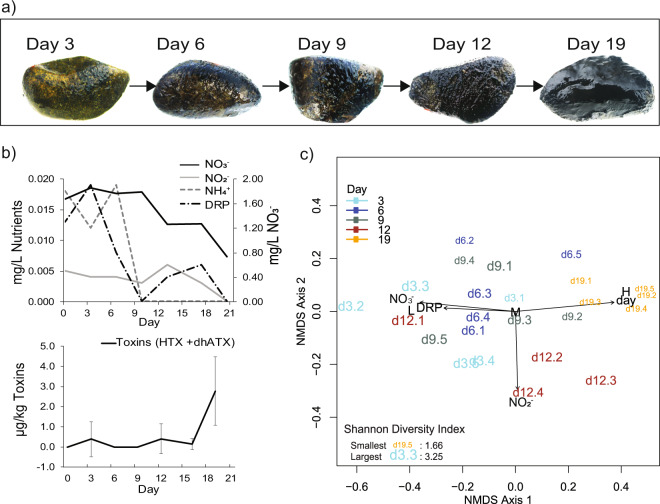
Images showing *Microcoleus* mat development, and plots showing changes in nutrients, toxins and mat communities over the 19-day proliferation event. **a** Photographs of biofilm-coated cobbles collected across five timepoints from day 3 to day 19, showing increasing biofilm cover. Biofilms are dark green in color, and the cobble surfaces are yellow. **b** Water column nutrient and biofilm toxin concentrations throughout the sampling period. Toxins were measured across six timepoints with four to eight replicates per time point. Day 0 indicates the start of the experiment (4th March 2016). Error bars = standard errors of means. Abbreviations: Nitrate (NO_3_^-^), Nitrite (NO_2_^-^), Ammonium (NH_4_^+^), Dissolved reactive phosphorus (DRP), Anatoxin-a (ATX), dihydro-anatoxin-a (dhATX). **c** NMDS ordination of amplicon data based on Bray-Curtis dissimilarities. Samples are labeled according to the day of collection with replicate number and the font size represented the Shannon diversity index. The final stress value is 0.14 and the vector indicates fitted environmental parameters significantly correlated to NMDS coordinates (*p* < 0.05, permutations = 999). Letters in black font indicate categorical vectors for *Microcoleus* relative abundance: low (L, 0 – 33%), medium (M, 33–67%), and high (H, 67–100%).

During the cyanobacterial proliferation, biofilm thickness and coverage visibly increased from thin and patchy to thick and complete coverage of the upper surfaces of submerged cobbles (Fig. 1a). Temperatures ranged from 14.6 to 23.3 °C, and biofilms received 12–14 h of daylight per day (Fig. S1). The river discharge was fairly stable (0.4 m^3^ s^−1^ to 0.9 m^3^ s^−1^) throughout the sampling period, which has been shown to be optimal for *Microcoleus* proliferations [22]. Consistent with previous findings [15], the initial proliferation stage coincided with elevated DIN and low DRP concentrations, both of which decreased over the sampling period (Fig. 1b, c). The nitrate concentration at the start of the experiment was 1.67 mg L^−1^, and decreased over 2-fold throughout the study period (Fig. 1b).

### *Microcoleus* proliferation

**Fig. 2:**
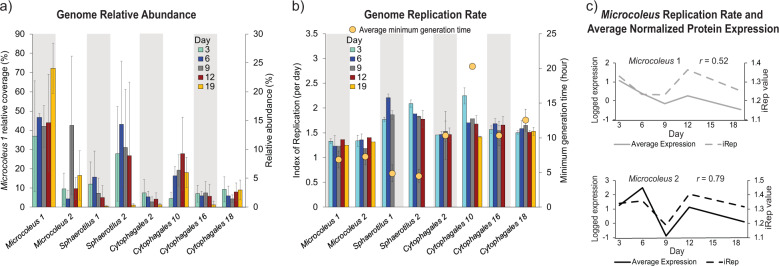
Plots showing genome coverage, predicted growth rates, and *Microcoleus* protein expression across five timepoints. **a** Average relative abundances of the eight dominant MAGs. *Microcoleus* 1 genome abundance is on a separate y-axis (left) due to higher abundance. Coverage values shown are relative to all the MAGs recovered in the dataset, and for the eight dominant MAGs sum to 62.51% at day 3, 78.61% at day 6, 80.02% at day 9, 72.80% at day 12, and 87.81% at day 19. Error bars = standard errors of means. **b** Barplot showing predicted replication rates for the same eight MAGs determined using iRep. Yellow circles indicate the average minimum generation time for each MAG determined using growthpred based on codon usage bias. **c** Close relationship between *Microcoleus* replication rate and per genome average log-transformed protein expression. The Pearson’s correlation coefficient (*r*) values are indicated.

Amplicon and genome relative abundance data revealed a gradual temporal shift in bacterial community structure (no archaea were detected), and a decrease in microbial community alpha diversity throughout the sampling period (Fig. 1c, a; Tables S2 and S4). This shift was largely driven by increases in the relative abundance of *Microcoleus*, which already dominated early day 3 biofilms, but also continued to increase throughout the bloom (increasing from 40% to 78% of the community) [63]. Biofilms were persistently colonized by two abundant *Microcoleus* species (*Microcoleus* 1 and 2). On average, *Microcoleus* 1 was 8.6 times more abundant. Environmental selection for these species was evidently strong, and conditions were uniform enough to limit niches available to other mat-forming species [64], including anatoxin-producing species or strains. The coexisting *Microcoleus* pair grew asynchronously throughout the study period, as reflected by their relative abundance profiles (Fig. 2a), and each displayed distinct temporal protein expression patterns, which were closely mirrored by predicted replication rates (Fig. 2b, c). This observation is in line with the reported coupling of photoautotroph growth and protein expression [65]. Despite the temporal variations in predicted growth rates and the large difference in abundance between *Microcoleus* 1 and 2, the average index of replication was near identical for both (iRep index 1.3 ± 0.1 *s*). Although the *Microcoleus* replication rates were modest relative to other major biofilm taxa, their predicted minimum generation times were relatively low (6.86 h ± 1.21 *s* and 7.28 h ± 1.14 *s*, respectively) (Fig. 2b), indicating the capacity for faster replication, particularly by *Microcoleus* 1.

### Phylogenetic comparison of *Microcoleus* 1 and 2

**Fig. 3:**
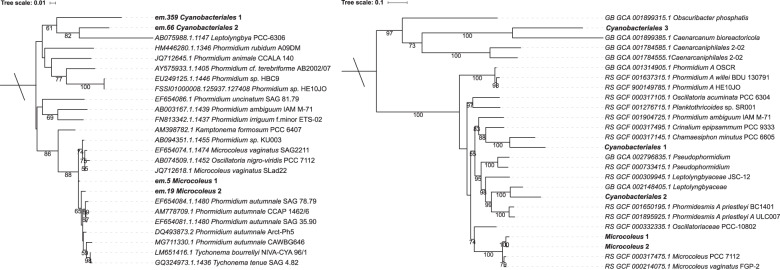
Maximum likelihood trees showing the phylogenetic relationship between biofilm cyanobacteria and other cyanobacteria. **a** Phylogenetic tree of cyanobacterial full-length 16S rRNA genes. EMIRGE-reconstructed 16S rRNA genes from this study are indicated in bold. The tree is rooted to *Escherichia coli* (A14565.1). **b** Tree based on 88 concatenated protein markers genes. The five biofilm cyanobacterial genomes are indicated in bold. The tree is rooted to GTDB *Gammaproteobacteria* UBA1515 (GCA_002323935.1). Scale bars represent number of substitutions per site. Bootstrap values over 50% are shown.

Two assembled full length 16S rRNA genes, and 88 concatenated marker gene protein sequences, from *Microcoleus* 1 and 2 formed highly conserved clades with *Microcoleus* species (Fig. 3, details in Supplementary Results). The 16S rRNA gene sequence similarity between the *Microcoleus* MAGs was 98.9% (Table S5), which is at the species threshold of 98.7–99%, [66]. In contrast, the genomes share only ~91% ANI over an alignable fraction (AF) of ~88% genes shared (Fig. S2; Table S6). Most intraspecies pairs share greater than 96.5% ANI and 60% of genes (as the AF), and these values have therefore been recommended as minimum thresholds for species discrimination [67]. Based on proximity to the 16S rRNA threshold, and a low genome ANI and AF, the two dominant *Microcoleus* in this study likely represent distinct species.

### Anatoxins and associated genes

While very toxic *Microcoleus* mats can contain hundreds of milligrams of anatoxins per kilogram [2], we detected only low concentrations (0 to 5.8 µg kg^−1^) of homoanatoxin-a and dihydroanatoxin-a (Fig. 1b). Toxin occurrence was patchy among replicates until the final time point, when the highest concentration (4.6 µg kg^−1^) was observed. Dihydrohomoanatoxin-a and anatoxin-a were below detection. No genes known to be associated with anatoxin synthesis were present within the metagenomes. Amplicon data indicate that 156 out of 186 cyanobacterial OTUs belonged to *Microcoleus* sp., and the remaining 30 OTUs were not from any known anatoxin-producers [68], suggesting toxins were produced by one or more of the rare *Microcoleus* species (Table S4) [18, 22].

### Temporal succession of the benthic mat community

**Fig. 4:**
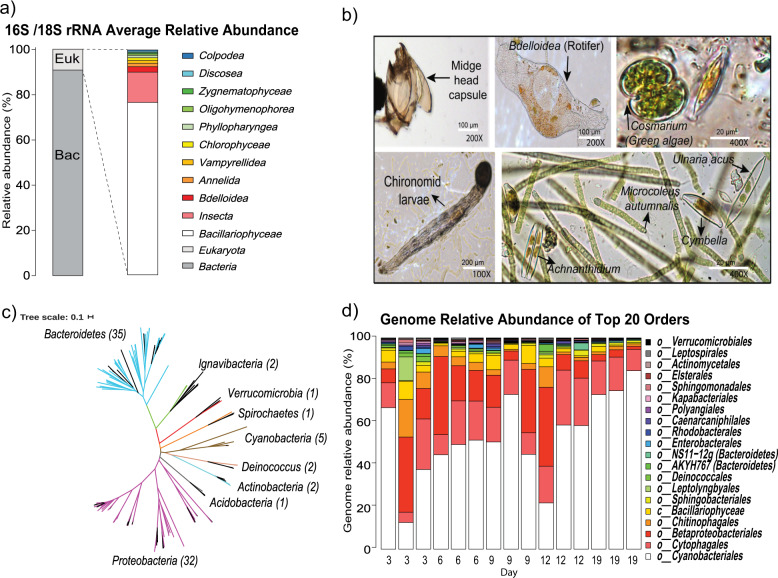
Community composition of *Microcoleus*-dominated mats. **a** Taxa abundances in the combined dataset based on EMIRGE-reconstructed 16S and 18S rRNA gene sequences. Bac Bacteria, Euk Eukaryota. **b** Microscopy images of *Microcoleus*, eukaryotic phototrophs (diatoms and *Cosmarium*), and biofilm grazers (chironomid larvae and rotifers). **c** Unrooted maximum likelihood tree of all 81 prokaryotic MAGs. Black lines indicate references from GTDB, and other tip colors indicate phyla-level clades. **d** Bar chart showing temporal changes in the relative genome coverage of the top 20 MAGs clustered by order level.

Ribosomal RNA genes assembled from WGS data and microscopy based observations confirm that submerged cobbles were colonized by a mixture of bacteria, diatoms, non-biting midge larvae (chironomid larvae are known to prey on diatoms [69]), and bdelloid rotifers (Fig. 4a, b; Table S7). Although *Microcoleus* overwhelmingly dominated the biofilms, the recovery of 81 MAGs spanning 9 bacterial phyla highlights the diversity of associated taxa (Fig. 4c). The most abundant non-mat forming members of the early biofilm community were *Betaproteobacteria* (particularly *Sphaerotilus*), followed by other cyanobacteria (*Leptolyngbyales* and *Candidatus* Caenarcanum), *Bacteroidetes* (notably *Cytophagales* and *Chitinophagales*), and diatoms (*Bacillariophyta*) (Fig. 4d and Table S2). Of these, *Betaproteobacteria*, *Bacillariophyta*, and mostly especially *Bacteroidetes*, have been shown to be major constituents of *Microcoleus* biofilms [18, 63, 70]. However, we found only a few *Bacteroidetes* (*Bacteroidetes* 5, *Chitinophagales* 3, *Cytophagales* 7 and 10) and *Alphaproteobacteria* (*Rhodobacterales* 2 and *Rhodoferax* 1) were positively correlated with *Microcoleus* (genome coverage *r* > 0.5; Fig. S3). The genome coverage of diatoms and most bacteria were negatively correlated with *Microcoleus* (*r* < −0.5; Fig. S3), although the predicted growth rates for some bacteria remained stable (i.e., *Cytophagales* 2, 16 and 18; Fig. 2b). *Betaproteobacteria* are often found in high abundance in stream biofilms [71], and likely represent a major part of the original, pre-*Microcoleus* proliferation, riverbed community in this study. While highly abundant in the early biofilms, *Betaproteobacteria* dropped seven-fold in relative genome coverage over the study period (from 18.7% ± 14.9 s to 2.6% ± 1.0 s) (Table S2), similar to previous observations by Brasell et. al. [63]. The large magnitude of decrease far outstripped the proportional increase in *Microcoleus* (only a two-fold increase), and betaproteobacterial abundance also decreased relative to other bacteria (Fig. S4). In addition, the replication rate of the most dominant betaproteobacterium declined through time (Fig. 2), suggesting betaproteobacterial growth declined during biofilm development.

### Biofilm organic matter degradation

Diverse taxa in the *Proteobacteria*, *Verrucomicrobiales*, *Spirochaetes*, *Deinococcales*, and *Actinobacteria*, were found to harbor genes for cellulose degradation, whereas most *Ignavibacteria* and *Bacteroidetes* were equipped with both cellulase and chitinase genes (Table S8). Proteomic data indicate *Bacteroidetes* (*Chitinophagales* 5 and 9) were engaged in biofilm-derived organic matter degradation through the expression of hydrolases and asparaginase (Tables S9 and S10). Their expression (following normalization against genome coverage) was higher in mature biofilms, indicating a higher rate of substrate hydrolysis and proteolytic activity as biomass increased. In addition, *Myxococcales* 1 encoded and expressed degradative enzymes, including proteases and ribonucleases (Tables S8, S9, and S10), and the greatest expression correlated with the peak abundance of *Microcoleus* 2 on day 12. *Myxococcales* are known to prey on other microorganisms (including the closely related cyanobacterium *Phormidium luridus*) and control cyanobacterial populations by lysing polysaccharides and other macromolecules in their trichomes and cell walls [72, 73]. It is therefore likely that *Myxococcales* 1 targeted *Microcoleus* for nutrients.

### Large protein investment associated with photosynthetic activity

**Fig. 5:**
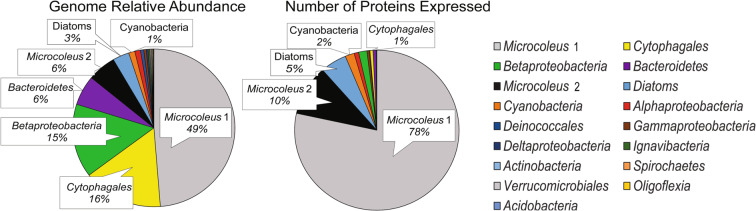
Comparison between genome relative abundance and number of proteins expressed by the biofilm taxa. The colors denote taxa at a variety of different taxonomic levels that indicate the best classification.

With the exception of the photoautotrophs (*R*^2^ = 0.96), we found little correlation between the genome coverage of community members and number of proteins detected (heterotroph *R*^2^ = 0.20; Fig. 5 and S5). Almost all proteins detected (95% based on presence/absence) were from phototrophs (cyanobacteria and diatoms), which represented only 58.9% of the community based on genome relative abundance (Fig. 5). While genes related to photosynthesis (including photosystems, ATP synthases, and light harvesting complexes) were a relatively minor fraction of the total community gene pool (2.6%) (Fig. S6), proteins related to photosynthesis were the largest category detected (17.7%), closely followed by phototroph ribosomal proteins (16.3%). Prior research has shown that a large fraction of gene transcripts and proteins expressed by phototrophs are related to photosynthesis [74, 75]. Oxygenic photosynthesis requires a large investment in resources, both in terms of photosynthesis and carbon fixation, which places limitations on photoautotroph growth rates [76, 77]. Bacteria with relatively slow growth strategies, such as *Microcoleus* (Fig. 2b), potentially attain disproportionately larger cell volumes and cellular RNA and protein contents [78]. This could explain the large fraction of phototroph proteins detected, along with the relatively large size of trichome-sheathed *Microcoleus* cells measuring 3 µm in diameter (Fig. 4b).

### Phototroph diversity and temporal activity

**Fig. 6:**
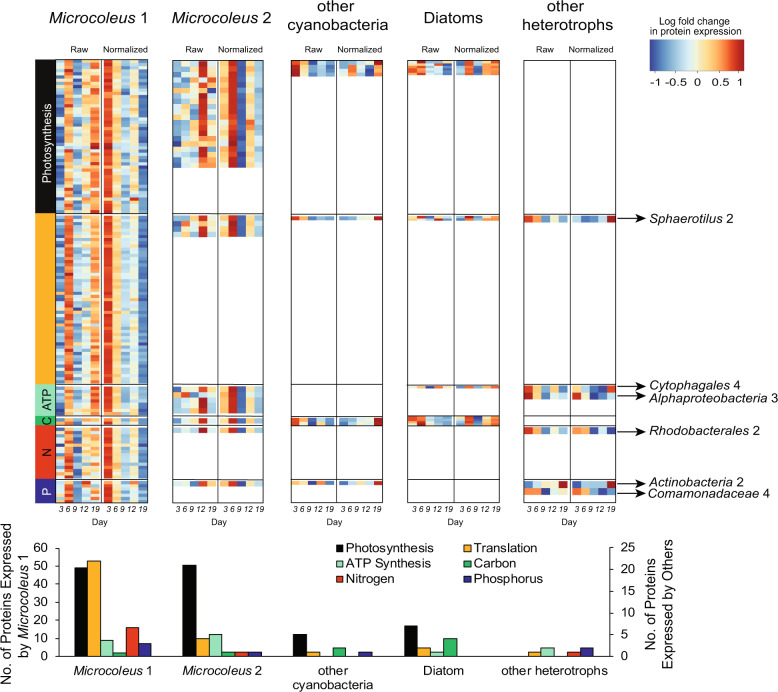
Protein expression in the biofilm communities. **a** Heatmaps of log-transformed protein expression data, including both unnormalized protein expression (raw) and protein expression normalized to genome coverage (normalized). Expression data are shown for major metabolic functions: photosynthesis, translation, ATP synthesis, Carbon fixation (C), Nitrogen metabolism (N), and Phosphorus (P). The arrows (right) indicate the heterotrophic taxa with proteins expressed. Protein data are scaled by row. Bar chart below shows the number of proteins expressed by each taxa group based on metabolic functions. *Microcoleus* 1 protein counts are shown on a separate y-axis (left).

Oxygenic phototroph contributions to biofilm primary production differed temporally. The majority of photosynthesis and carbon fixation related protein expression from *Microcoleus* 1 and 2 were higher at later times points (days 12 and 19, respectively), whereas expression from diatoms and other cyanobacteria dropped two-fold through time (Fig. 6 and Table S9). Protein contributions from most, but not all heterotrophs also decreased as biofilms matured. Despite this, when proteins were normalized to changes in taxon-specific genome abundance (i.e., expression per genome), these trends in phototroph expression reversed (Fig. 6 and Table S10). Therefore, although *Microcoleus* spp. contributed more to overall protein expression and photosynthesis as biomass increased, results suggest a decline in per cell energy capture over time. The decline cannot be attributed to a decrease in *Microcoleus* growth, as predicted replication rates remained stable - aside from a transient drop in growth rate and overall protein expression associated with high light conditions during day 9 (4.8 times higher than earlier or later; Fig. 2c and S1).

While we only detected activity for oxygenic photosynthesis, we also found genes indicative of anoxygenic photosynthesis (e.g., genes encoding BChl *a*, PufM, and LHC proteins; Table S8) [79] in *Acidobacteria* and *Alphaproteobacteria* (*Rhodobacterales* 1 and 2, and *Sphingomonadales* 2). No protein expression associated with anoxygenic photosynthesis was detected, although this may reflect peptide detection depth limitations. The acidobacterium was equipped with an inorganic carbon concentrating mechanism (CCM), which enhances inorganic carbon uptake in CO_2_-limited environments [80]. Although the anoxygenic photosynthetic activity might seem to be negligible, these data indicate that the biofilm community harbored the capacity to harvest light for ATP production using alternative electron donors. For example, *Sphingomonadales* use thiosulfate rather than H_2_O as electron donor for aerobic anoxygenic photosynthesis [81, 82]. These anoxygenic photoheterotrophs (except *Sphingomonadales* 2) were proportionally more abundant in early biofilms, and presumably persisted from pre-bloom conditions.

### Dissolved nitrogen acquisition by *Microcoleus* and other taxa

In line with previous studies of *Microcoleus* in New Zealand and Californian rivers [18, 21], *Microcoleus* 1 and 2 lack nitrogenase genes (Table S8). Key nitrogenase genes were also absent from the wider biofilm community. Results indicate that *Microcoleus* 1 dominated biofilm nitrogen acquisition throughout the proliferation by up-taking dissolved nitrate and urea (via NtrBCD and UrtA transporters, respectively, Tables S9 and S10). Although we did not measure urea (NH_2_CONH_2_), urea is a problematic agricultural pollutant of water bodies [83]. Both *Microcoleus* genomes harbored the same capacity for nitrogen acquisition, although protein expression by *Microcoleus* 2 was below detection for nitrogen metabolism (excluding glutamine synthase). Expression of *Microcoleus* 1 proteins associated with nitrogen metabolism corresponded strongly with temporal patterns of photosynthesis and carbon assimilation. While these were all more highly expressed during early biofilm formation, data indicate *Microcoleus* 1 was persistently active in nitrate transport (NtrBCD), assimilatory nitrite reduction (NirBD), urea uptake and degradation (UrtAC and UreABCD), nitrogen regulation and assimilation (P-II protein), and cyanophycin synthesis (CphA) and breakdown (CphB ×2) (Fig. 6).

**Fig. 7:**
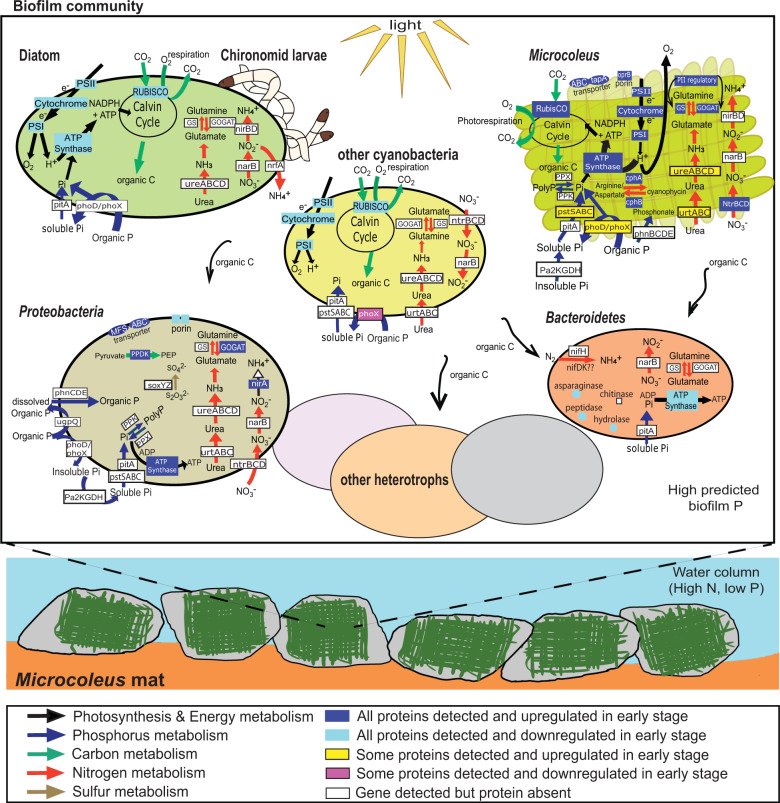
Schematic illustrating metabolic interactions among biofilm community members using functional genomics and proteomics. Schematic of key genes and proteins expressed within the *Microcoleus*-dominated biofilm, with expression up/down regulation shown relative to early/late stage growth of the biofilm. Gene and protein details are documented in Tables S8–S11.

Cyanophycin is common among cyanobacteria, enabling them to store nitrogen in environments subjected to fluctuating nitrogen supply [84]. Prior research has also shown that phosphate starvation promotes cyanophycin accumulation [85]. Both *Microcoleus* genomes were equipped with two cyanophycin gene clusters, *cph1* and *cph2*, each containing cyanophycin synthetase and cyanophycinase genes, similar to *Anabaena* sp. PCC 7120 [86]. Our proteomics data showed CphA (cyanophycin synthetase) and CphB (cyanophycinase) expression by *Microcoleus* 1 throughout biofilm growth (Tables S9 and S10), suggesting active storage and utilization of cyanophycin granules to cope with fluctuations in nutrient supply. Along with proteins for photosynthesis, carbon fixation and nutrient acquisition (Fig. 6), the expression levels of both cyanophycin synthetase and cyanophycinase decreased over time (after normalizing to genome coverage, Fig. 7). Previous studies have primarily focused on cyanophycin in diazotrophs, showing that cyanophycin is used by non-heterocystous cyanobacteria when unable to fix nitrogen during the daytime [84, 87], or that cyanophycin is stored at the polar regions of heterocysts to supply nitrogen to vegetative cells [88]. Further research is needed to verify the creation and use of cyanophycin in non-diazotrophic *Microcoleus* mats.

Various genes involved in nitrogen assimilation from diatoms, other cyanobacteria and diverse bacteria (*Proteobacteria*, *Bacteroidetes*, *Verrucomicrobia*, *Ignavibacteria*) inhabiting the biofilms were identified, including mechanisms for assimilatory nitrate and nitrite reduction, urea uptake and degradation, and nitrogen regulation (P-II protein and glutamate synthase) (Table S8). Genes for dissimilatory nitrate reduction to ammonium were also present, but only a handful of genes for the denitrification pathway. Given the relatively low protein contribution of heterotrophs in this study, we unsurprisingly detected the expression of only two proteins from this group related to nitrogen metabolism (Fig. 6, Tables S9 and S10).

### Phosphate acquisition

Phosphate is commonly limiting in freshwater systems [89]. *Microcoleus* biofilm communities were recently shown to harbor diverse mechanisms for phosphate acquisition [18]. Our genomic data agree, showing numerous members of the biofilm community possessed similar genes for low (*pitA*) and high affinity (*pstABCS*) inorganic phosphate (P_i_) transporters, organic phosphate mineralization (phosphate monoester metabolism via alkaline phosphatase genes, *phoA*, *phoD*, and *phoX*, and acid phosphatase gene, *aphA*; glycerol phosphate metabolism via glycerolphosphodiester phosphodiesterase gene, *ugpQ*), and in/organic phosphate metabolism and transport regulation (*phoBRU*) (Fig. 7). Further to this, we found that *Deinococcales* 1 and *Microcoleus* 1 and 2 harbor an additional mechanism for organic phosphate mineralization via a Class IIIA haloacid dehydrogenase (HAD) superfamily phosphatase (*yqeG*-like). Our data also indicate the widespread capacity for organic phosphate transport (phosphonate transport, *phnBDCE*) [90], phosphate storage via genes for polyphosphate synthesis (polyphosphate kinase, *ppk*) and degradation (exopolyphosphatase, *ppx*) [91, 92], and possible P_i_ solubilization via glucose dehydrogenase (*gcd*) or 2-ketoglucose dehydrogenase (*2kgdh*). Both *Microcoleus* 1 and 2 carry almost all of these mechanisms, enabling them to scavenge and utilize various forms of organic phosphate (*phnBDCE*, *phoD*, *phoX*, *ugpQ*, *yqeG*-like), solubilize P_i_ (*2kgd*), uptake dissolved P_i_ (*pitA*, *pstABCS*), and stockpile phosphate (*ppx*, *ppk*).

The putative P_i_ solubilization genes we identified resemble a recently described family of membrane-bound pyrroloquinoline quinone (PQQ) dependent 2-keto-D-glucose (2-ketoglucose) dehydrogenases (*2kgdh*) [93]. The *Pa*2KGDH enzyme produces 2-ketogluconic acid from 2-ketoglucose, derived from glucose via an unknown mechanism, and is related to membrane-bound and soluble glucose dehydrogenases, which produce gluconic acid (genes *gcd* and *gdhB*). Production of microbial gluconic acid, and more highly acidic 2-ketogluconic acid, by membrane-bound dehydrogenases can liberate P_i_ from calcium phosphates by acidification of the external environment [94]. We found that *Microcoleus* also possesses *oprB* genes for carbohydrate-selective porin proteins, which could facilitate the import of glucose, along with some other sugars [95] as fuel for P_i_ solubilization, or the import of organic phosphate compounds for direct P acquisition [96]. Both *Microcoleus* genomes lack the *pqq* operon for PQQ synthesis for self-supported PQQ-dependent *gdh*/*2kgdh* activity and phosphate solubilization. However, some biofilm *Proteobacteria* possessed *pqq* genes (*Sphaerotilus* 2, *Alphaproteobacteria* 1, and *Myxococcales* 1 and 2), and could synthesize PQQ as a public good. Reliance on exogenous PQQ for GDH/2KGDH is a known strategy of some bacteria [97, 98]. While *Microcoleus* might be able to extract P_i_ from particulates with community assistance, proteins expressed by these genes (PQQ or PQQ-dependent GDH/2KGDH) were not detected in this study.

Despite diverse and widespread mechanisms for phosphate metabolism within the biofilm community, most of the measured proteins were expressed by *Microcoleus* 1 (high affinity phosphate transporters, PstBS and PstS x2 encoded from three distinct operons; organic phosphate phosphatases, PhoX and YqeG-like) (Fig. 6; Tables S9 and S10). Results indicate that *Microcoleus* 1 actively acquired dissolved P_i_ and metabolized organic phosphate (e.g., phosphate monoesters) (Fig. 7). Coexpression of phosphatases suggests *Microcoleus* 1 supplemented its exogenous P_i_ intake by hydrolyzing organic phosphate, such as dissolved phosphate monoesters, either periplasmically [99] or extracellularly [100]. In addition, *Microcoleus* 1 expressed proteins from its two *oprB* genes (described above), which could have facilitated the uptake of organic phosphate compounds. Overall, results indicate the proliferation was in part sustained by organic phosphate derived from the biofilm community, and *Microcoleus* did not solely rely on dissolved P_i_.

While we predict that DRP was elevated within the sampled biofilms due to P_i_ liberation from trapped sediments under the high daytime pHs associated with photosynthetic activity [24], we found that both *Microcoleus* 1 and 2 expressed the high affinity, low velocity phosphate specific transporter system (Pst), while expression of the higher velocity PitA proteins were not detected. This suggests *Microcoleus* experienced phosphate limitation. Pst expression increases as phosphate becomes increasingly scarce [101], and has been shown to be active when DRP concentrations are as low as 5 μg L^−1^ in marine [102] and 50 μg L^−1^ in lake [103] environments, encompassing the DRP concentrations we measured in the water column (~15 μg L^−1^, Fig. 1). PstS is shown to be repressed at DRP concentrations above ~0.6 mg L^−1^ [104], which is marginally higher than the elevated DRP concentrations measured by Wood et al. [24] within thick photosynthesizing *Microcoleus* mats. Duplicate *pst* clusters in cyanobacteria lacking *pitA* have been shown to favor distinct P_i_ ranges to cater for starvation and replete conditions [105]. As *Microcoleus* has higher velocity *pitA*, the duplicate *pst* operons of *Microcoleus* instead might vary in their capacity for P_i_ accumulation under limiting conditions [106]. The high-affinity phosphate uptake system also appears to play a critical role in biofilm formation [107].

## Conclusions

The benthic mats were dominated by two stably coexisting *Microcoleus* species. The majority of proteins detected derived from cyanobacteria and diatoms, which can be accounted for by the large resource investment needed for photosynthesis and carbon fixation. *Microcoleus* employed diverse strategies for acquiring nutrients throughout mat development. Proteogenomics data indicate that *Microcoleus* actively sourced nitrogen via urea and nitrate uptake, and suggest that *Microcoleus* utilized cyanophycin for internal carbon and nitrogen storage. Our data show *Microcoleus* is equipped with multiple mechanisms for P_i_ transport, and both dominant species possessed three mechanisms for organic phosphorus mineralization. Furthermore, *Microcoleus* actively expressed proteins to simultaneously acquire P_i_ and organic phosphorus. Genomic data suggests *Microcoleus* could potentially carryout P_i_ solubilization with the help of collaborative PQQ producers also present in the mats. Results of this study provide insights into how *Microcoleus* species grow under low P_i_ conditions, and suggests that both inorganic and organic phosphorus are important nutrient sources for *Microcoleus* in benthic biofilms.

## Supplementary information

Supplementary materials

Supplementary tables
