# Supplementary material for: Tools for successful proliferation: diverse strategies of nutrient acquisition by a benthic cyanobacterium
Source: ISME J. 2020 May 18;14(8):2164–78. doi: 10.1038/s41396-020-0676-5 (PMC7367855; doi:10.1038/s41396-020-0676-5)
Supplement: Supplementary file 1 — Supplementary materials [file 41396_2020_676_MOESM1_ESM.pdf]

# Supplementary Materials

## Supplementary Methods

**Eukaryotic genome binning.** Eukaryotic bins were identified by first assigning taxonomy to all unbinned contigs with Kraken v1.0 [1] using the MiniKraken DB_8GB database (downloaded June 7, 2018). Contigs classified as eukaryotic were extracted then dereplicated using MUMmer v3.23. Contigs that fell completely within the bounds of a longer contig and possessed with 100% sequence similarity were removed from the dataset. In cases where partial overlap between contigs was observed, the shorter contig was removed from the dataset if the region of overlap matched this similarity threshold, and the degree of overlap was greater than 10% of the smaller contigs length. Diatom bins were created from this final contig set by extracting only those contigs at least 10 kbp in length with a phylum-level classification of *Bacillariophyta*. Tetranucleotide frequencies and coverage values for each contig were computed, then centered and scaled using the Box-Cox transformation in R using the caret library. Clusters of contigs ('primary bins') were identified using hierarchical clustering with Ward's clustering criterion [2]. The optimal number of clusters in the data was estimated using the gap statistic. Shorter contigs were recruited to the primary bins by constructing a random forest classifier with 1 000 decision trees in scikit-learn v0.19.1 [3]. Model training was performed using 10-fold cross validation, whereby 75% of binned contigs were used as the training set, with proportional representation from each primary bin.

To objectively determine the confidence threshold at which shorter reads could be reliably assigned to one of the primary bins, all contigs using in model training were randomly cut into fragments between 1 500 and 2 500 base pairs long. Tetranucleotide frequencies and coverage values for each fragment were then recalculated, and each fragment classified using the random

forest model. The confidence of each fragment classification was expressed as the proportion of individual decision trees supporting the most common assignment, and assignments were considered correct if the fragment was assigned to the same primary bin as the contig from which it originated. Inspection of these data revealed that approximately 13% of read fragments obtained confidence scores of at least 0.5, which yielded 97% assignment accuracy. We then classified *Bacillariophyta* contigs that were not included in the original binning round (i.e. those under 10 kbp in length) to the primary bins, requiring a support confidence of at least 0.5. Finally, all binned contigs were checked against the prokaryotic MAGs to ensure that they, or contigs that had been removed in the MUMmer process, had not been binned to one of the prokaryotic MAGs. Five such contigs, from 6 402 contigs in the *Bacillariophyta* bins, were observed to have been assigned to prokaryotic MAGs and were subsequently removed.

**Whole genome comparisons.** Genome sequences related to *Microcoleus* were retrieved from NCBI genome database [4]. Reciprocal BLASTN and BLASTP [5] were carried out for all genomes to calculate the average nucleotide identity (ANI) [6]. Sequences were considered orthologous on the basis of reciprocal BLASTP analysis, if they had a minimum bit score of 60, an alignment length of >70%, and shared identity of >30%. A matrix of whole genome BLAST-based similarity scores were generated and UPGMA tree was constructed with the Phangorn package [7] using R version 3.5.1 [8]. Correlation analysis was analyzed by the Spearman method using the corrplot function in R.

**Peptide detection via LC-MS/MS.** Peptides were loaded onto a 0.3 x 10 mm trap column packed with Reprosil C18 media (Dr Maisch, Ammerbuch, Germany) and desalted for 6 min at 2 μL $min^{-1}$ before being separated on a 0.075 × 200 mm picofrit column with a 15 μm integrated electrospray emitter (New Objective, MA, USA) that was packed in-house with Reprosil C18 media. Solvents for HPLC were 0.1% formic acid (buffer A) and 0.1% formic acid in acetonitrile (buffer B). Separations were performed at a flow rate of 250 nL $min^{-1}$ using a gradient of: 2 min at 1% B; 103 min from 1% B to 35% B; 5 min from 35% B to 98% B; 5 min at 98% B; 1 min from 98% B to 1% B; 9 min at 5% B. For MS/MS analysis an initial MS survey scan from 350 – 1 600 m/z was collected for 250 ms, followed by MS/MS scans of 80 – 1 600 m/z and 40 ms for the 40 most abundant precursor ions detected with a charge state of between 2 and 5. Previously selected precursor ions were excluded for 30 s.

## Supplementary Results

**Phylogenetic comparisons of *Microcoleus* 1 and 2 to other cyanobacteria.** Two assembled full length 16S rRNA genes from the dominant bloom-forming cyanobacterial pair (*Microcoleus* 1 and 2) formed a highly conserved clade with *Phormidium autumnale*, *Microcoleus vaginatus*, *Tychonema bourrelly*, *Tychonema tenue* and *Oscillatoria nigro-viridis* species (Fig. 3a and Table S5). Moreover, due to a high level of morphological and phylogenetic similarity, Strunecký et al. [9] proposed renaming *P. autumnale* to *Microcoleus autumnalis*. *Microcoleus* 1 and 2 each share the closest sequence similarity with cf. *Tychonema* sp. SAG 2388 (99.1%) and *Phormidium autumnale* SAG 35.90 (99.2%), respectively – similarities generally indicative of species-level identity. In agreement with rRNA phylogeny, our analysis of 88 concatenated marker gene protein sequences show that the two bloom-formers share a closer evolutionary relationship with *Microcoleus* species than to other *Phormidium* species (Fig. 3b). This conclusion is also supported by high pairwise ANI of >85% between the pair and *Microcoleus sp.* PCC 7112 (*Oscillatoria nigro-viridis)* and *M. vaginatus* [6]. *M. vaginatus* is the type species of *Microcoleus*, and is a cosmopolitan cyanobacterial species with broad environmental tolerances and distributions [9]. *M. vaginatus* spp. have been found in terrestrial habitats ranging from polar regions to hot deserts, and inhabit aquatic environments and soils. *M. autumnalis* species have a similarly broad distribution – they have also been found in both soil and aquatic terrestrial environments, and in polar to temperate habitats [10]. While each *Microcoleus* pair from this study share 88-89% ANI with *M. vaginatus* spp., they only share 70% to 80% ANI similarity with other *Phormidium* species (Fig. S2, Table S5). Three other cyanobacterial genomes (*Cyanobacteriales* 1 to 3) recovered from the mats were more closely related to *Synechococcales* (*Chamaesiphon minutus* and *Leptolyngbyaceae*) and *Candidatus Caenarcanum bioreactoricola* (Fig. 3b).

## Supplementary Figures

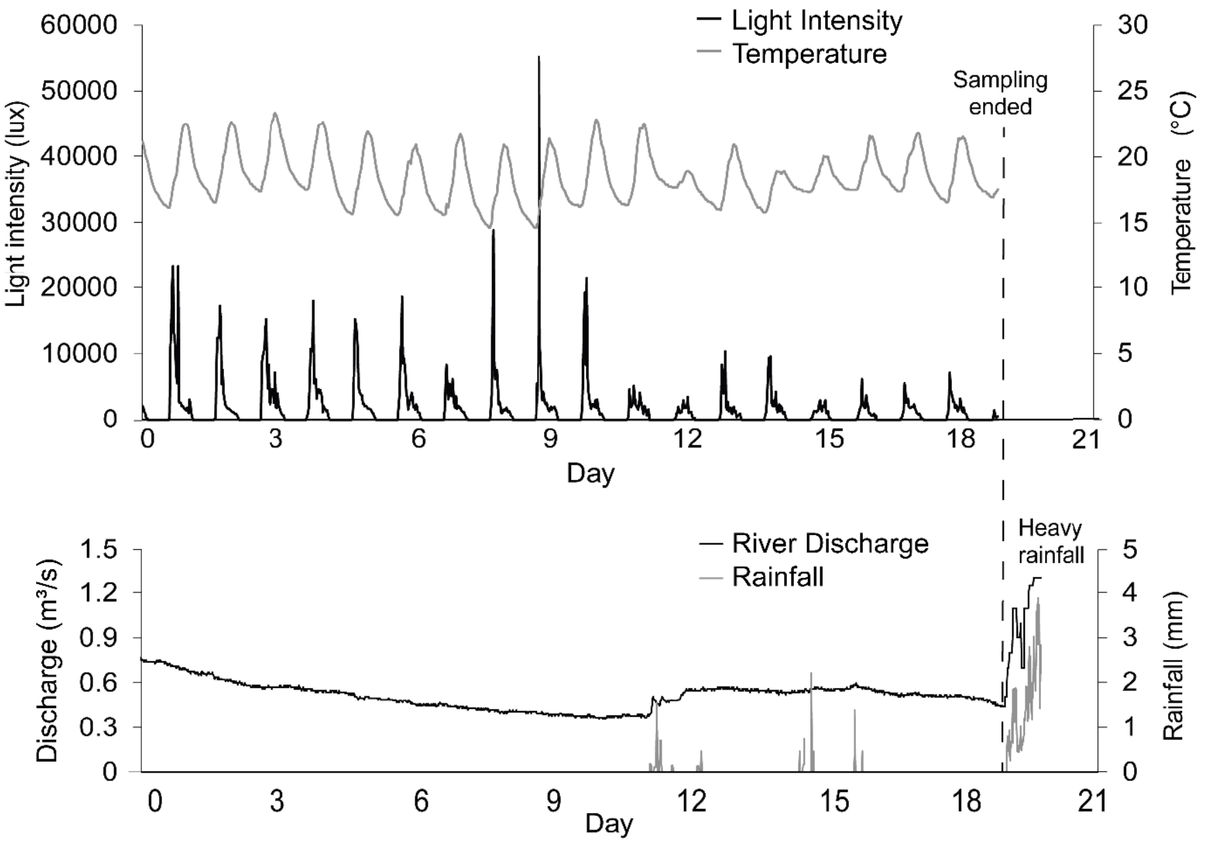


Figure S1. Light, temperature, river discharge and rainfall data across the sampling period. Day 0 indicates the start of the experiment (4th March 2016). The range of daily high and low water temperatures across the sampling period were 18.6-23.3 °C (daily highs) and 14.6-17.7 °C (daily lows). Dotted vertical line indicates end of sampling at day 19 due to a period of heavy rainfall afterwards. The spike of light intensity at day 9 (4.8 times higher than earlier or later intensities) corresponded to decreased protein expression.

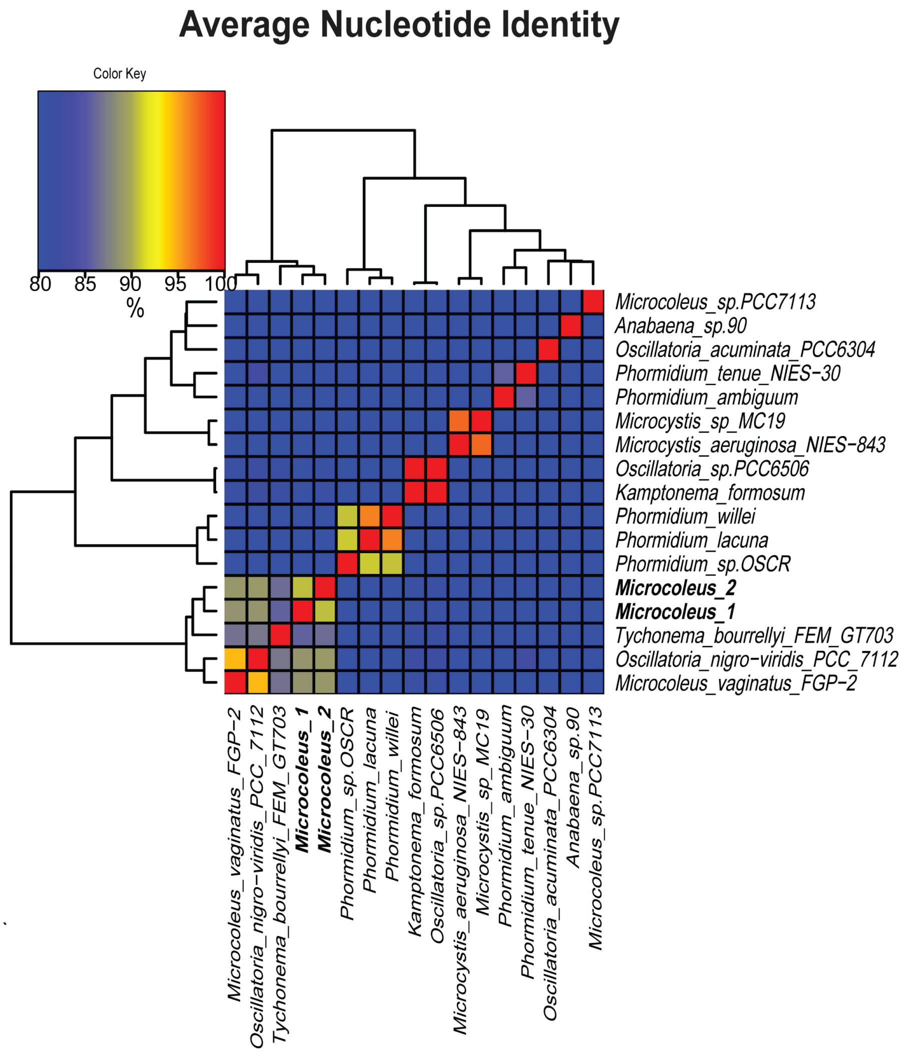


Figure S2. Genome relatedness of the dominant *Microcoleus* pair (in bold), along with 15 closely-related species retrieved from NCBI genome database (Table S4). *Oscillatoria nigro-viridis* PCC 7112 (GCA_000317475.1) is classified as *Microcoleus* sp. in GTDB. Heatmaps show pairwise percent average nucleotide identity (ANI). Hierarchical clustering was done based on Ward's algorithm. Color gradient indicates percent similarity.

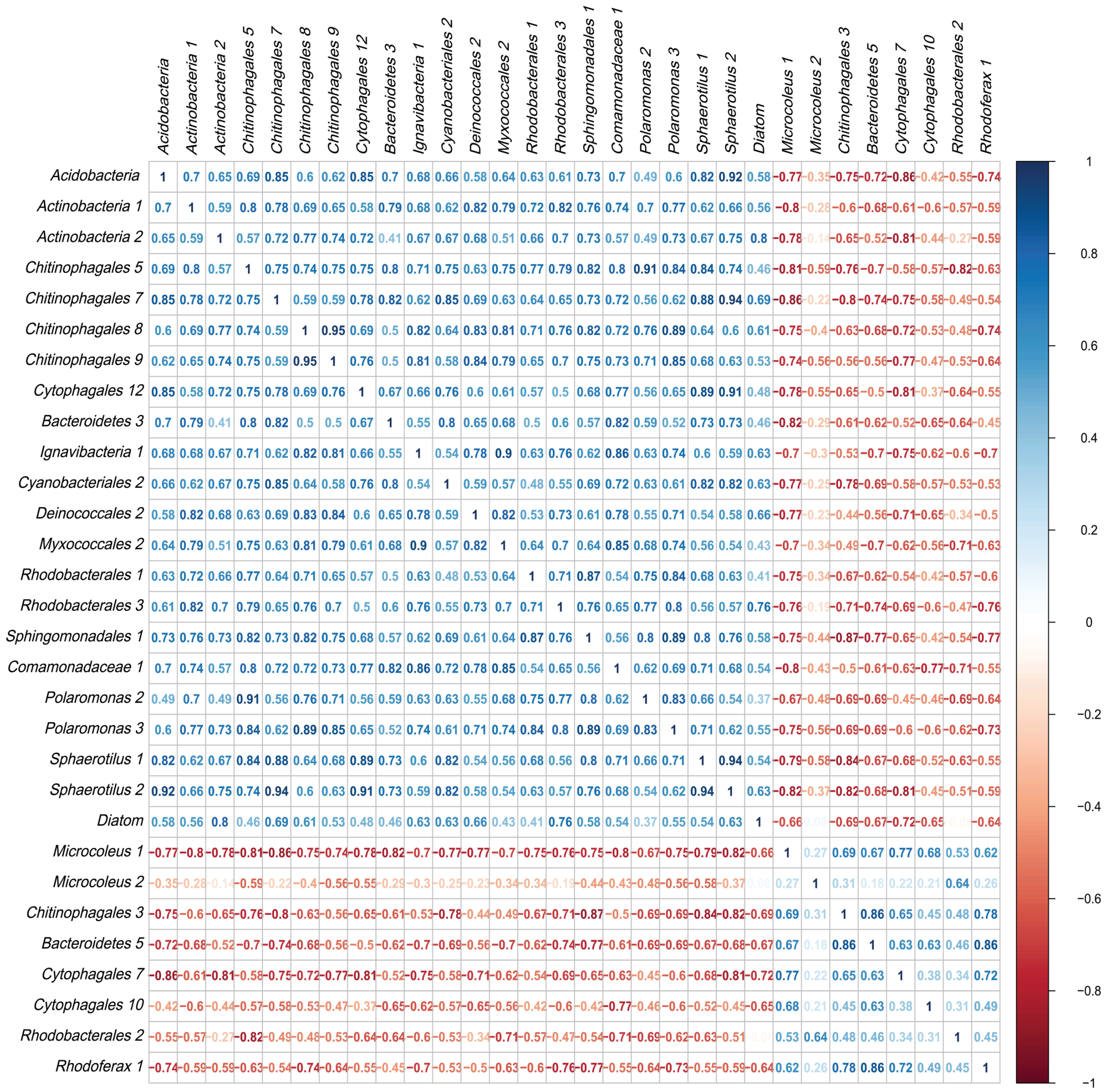


Figure S3. Spearman correlations of genome coverage with $r > 0.5$ or $r < -0.5$ between *Microcoleus* and other biofilm members. Color gradient indicates correlation value.

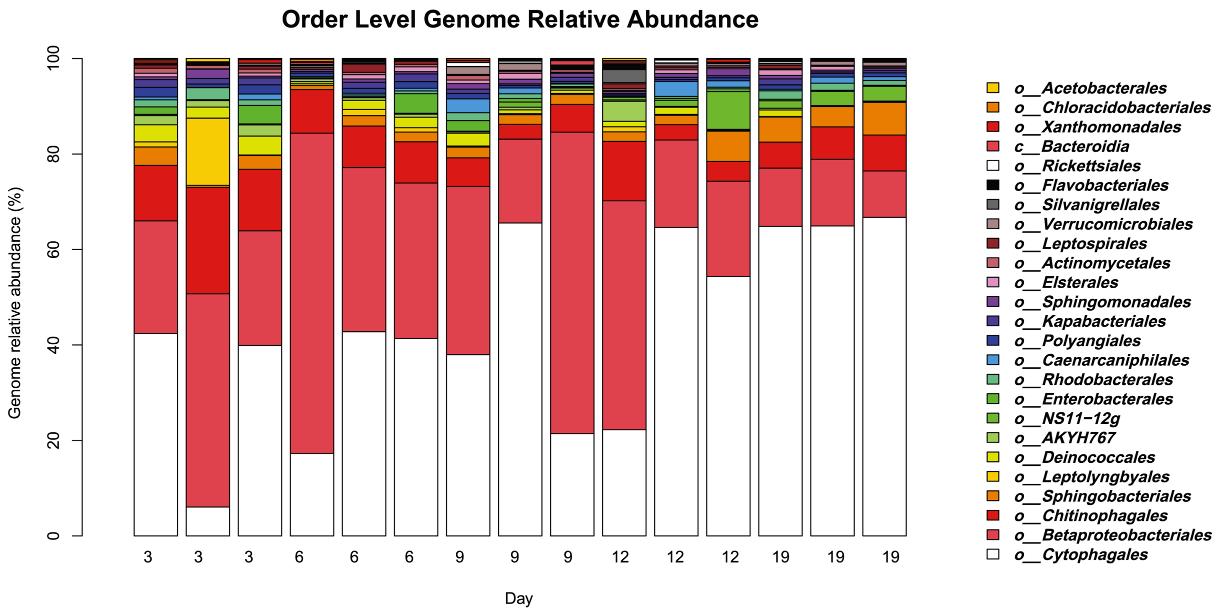


Figure S4. Bar plot showing temporal changes in the relative genome coverage of all taxa, excluding cyanobacteria and diatoms, clustered by order level.

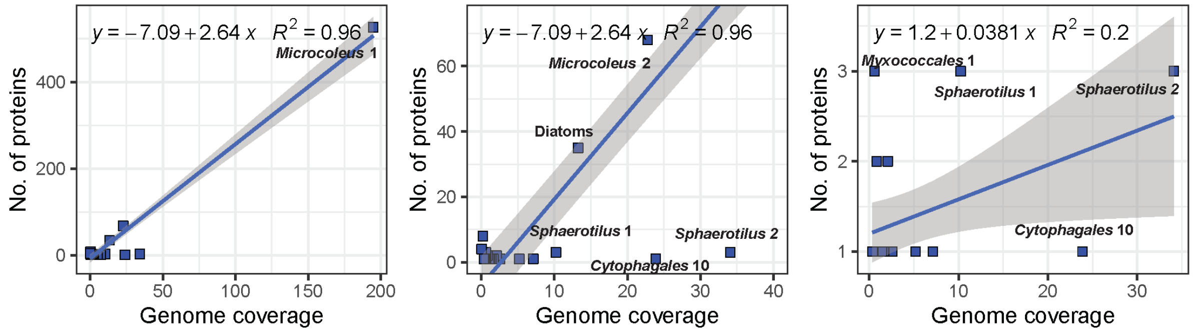


Figure S5. Relationship between average genome coverage and number of proteins expressed by individual taxa in the biofilm community. Linear regression line is indicated on the graph with associated $R^2$ value. Plots were generated using ggplot2 [11]. The leftmost plot shows proteins detected for all taxa, and the correlation between genome coverage and number of proteins detected per MAG. The middle plot is an inset of the leftmost plot, showing all taxa other than *Microcoleus* 1. Most of the heterotrophic taxa fall below the line, indicating low protein expression relative to genome abundance. The rightmost plot shows the correlation between genome coverage and proteins detected for taxa, excluding all dominant phototrophs.

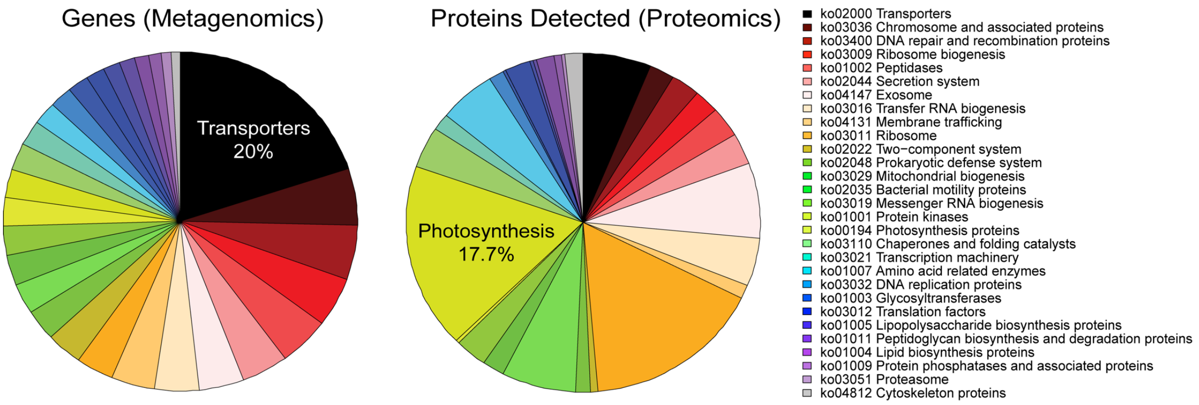


Figure S6. Functional potential and protein expression within the biofilm community. Annotated genes and proteins were classified based on KEGG BRITE functional hierarchies.

**Supplementary References**


1. Wood DE, Salzberg SL. Kraken: ultrafast metagenomic sequence classification using exact alignments. *Genome Biol* 2014; **15**: R46.
2. Ward JH. Hierarchical grouping to optimize an objective function. *J Am Stat Assoc* 1963; **58**: 236–244.
3. Pedregosa F, Varoquaux G, Gramfort A, Michel V, Thirion B, Grisel O, et al. Scikit-learn: Machine learning in Python. *J Mach Learn Res* 2011; **12**: 2825–2830.
4. NCBI Resource Coordinators. Database resources of the National Center for Biotechnology Information. *Nucleic Acids Res* 2018; **46**: D8–D13.
5. Altschul SF, Madden TL, Schäffer AA, Zhang J, Zhang Z, Miller W, et al. Gapped BLAST and PSI-BLAST: a new generation of protein database search programs. *Nucleic Acids Res* 1997; **25**: 3389–3402.
6. Konstantinidis KT, Tiedje JM. Towards a genome-based taxonomy for prokaryotes. *J Bacteriol* 2005; **187**: 6258–6264.
7. Schliep K, Potts AJ, Morrison DA, Grimm GW. Intertwining phylogenetic trees and networks. *Methods Ecol Evol* 2017; **8**: 1212–1220.
8. R Core Team. R: a language and environment for statistical computing. 2018. R Foundation for Statistical Computing, Vienna, Austria.
9. Strunecký O, Komárek J, Johansen J, Lukešová A, Elster J. Molecular and morphological criteria for revision of the genus *Microcoleus* (*Oscillatoriales, Cyanobacteria*). *J Phycol* 2013; **49**: 1167–1180.
10. Chrismas NAM, Anesio AM, Sánchez-Baracaldo P. Multiple adaptations to polar and alpine environments within cyanobacteria: a phylogenomic and Bayesian approach. *Front Microbiol* 2015; **6**: 1–10.

11. Wickham H. ggplot2: Elegant graphics for data analysis. 2016. Springer-Verlag, New York.
